# Effect of 2,6-Bis-(1-hydroxy-1,1-diphenyl-methyl) Pyridine as Organic Additive in Sulfide NiMoP/γ-Al_2_O_3_ Catalyst for Hydrodesulfurization of Straight-Run Gas Oil

**DOI:** 10.3390/molecules22081332

**Published:** 2017-08-15

**Authors:** Carlos Eduardo Santolalla-Vargas, Victor Santes, Erick Meneses-Domínguez, Vicente Escamilla, Agileo Hernández-Gordillo, Elizabeth Gómez, Felipe Sánchez-Minero, José Escobar, Leonardo Díaz, Oscar Goiz

**Affiliations:** 1 Departamento de Biociencias e Ingeniería, Centro Interdisciplinario de Investigaciones y Estudios sobre Medio Ambiente y Desarrollo (CIIEMAD), Instituto Politécnico Nacional, C.P. 07340 Ciudad de México, Mexico; csantolallav@ipn.mx (C.E.S.-V.); iqi_erick@hotmail.com (E.M.-D.); vescamillar@ipn.mx (V.E.); ogoiza@ipn.mx (O.G.); 2 Instituto de Investigaciones en Materiales, Universidad Nacional Autónoma de México, Circuito Exterior S/N, Ciudad Universitaria, Coyoacán, C.P. 04510 Ciudad de México, Mexico; agileohg@iim.unam.mx; 3 Instituto de Química, Universidad Nacional Autónoma de México, Circuito Exterior S/N, Ciudad Universitaria, Coyoacán, C.P. 04510 Ciudad de México, Mexico; eligom@iquimica.unam.mx; 4 Departamento de Ingeniería Química Petrolera, ESIQIE, Instituto Politécnico Nacional, Zacatenco, C.P. 07738 Ciudad de México, Mexico; jfsanchez@ipn.mx; 5 Instituto Mexicano del Petróleo, Eje Central Lázaro Cárdenas 152, San Bartolo Atepehuacan, Gustavo A. Madero, C.P. 07730 Ciudad de México, Mexico; jeaguila@imp.mx (J.E.); ldiazg@imp.mx (L.D.)

**Keywords:** hydrodesulfurization, organic additive, NiMoP, sulfided catalysts

## Abstract

The effect of 2,6-bis-(1-hydroxy-1,1-diphenyl-methyl) pyridine (BDPHP) in the preparation of NiMoP/γ-Al_2_O_3_ catalysts have been investigated in the hydrodesulfurization (HDS) of straight-run gas oil. The γ-Al_2_O_3_ support was modified by surface impregnation of a solution of BDPHP to afford BDPHP/Ni molar ratios (0.5 and 1.0) in the final composition. The highest activity for NiMoP materials was found when the molar ratio of BDPHP/Ni was of 0.5. X-ray diffraction (XRD) results revealed that NiMoP (0.5) showed better dispersion of MoO_3_ than the NiMoP (1.0). Fourier transform infrared spectroscopy (FT-IR) results indicated that the organic additive interacts with the γ-Al_2_O_3_ surface and therefore discards the presence of Mo or Ni complexes. Raman spectroscopy suggested a high Raman ratio for the NiMoP (0.5) sample. The increment of the Mo=O species is related to a major availability of Mo species in the formation of MoS_2_. The temperature programmed reduction (TPR) results showed that the NiMoP (0.5) displayed moderate metal–support interaction. Likewise, X-ray photoelectron spectroscopy (XPS) exhibited higher sulfurization degree for NiMoP (0.5) compared with NiMoP (1.0). The increment of the MoO_3_ dispersion, the moderate metal–support interaction, the increase of sulfurization degree and the increment of Mo=O species provoked by the BDPHP incorporation resulted in a higher gas oil HDS activity.

## 1. Introduction

The pollution is a problem in the society today. In this sense, environmental legislations are more rigorous with contaminants in diesel fuel, and the sulfur content in diesel is about 10 ppm [1]. The production of ultra-low sulfur diesel is of paramount importance in the hydrotreatment process. Therefore, the hydrodesulfurization (HDS) catalysts have to be more efficient to satisfy the environmental requirements. Conventional catalysts consist of molybdenum supported over alumina with either cobalt or nickel as promoters [2]. These catalysts have shown interesting performance in HDS reactions of sulfur model molecules [3,4]. However, the Co (Ni) promotion and dispersion of MoS_2_ (WS_2_) have to improve for increasing the catalytic activities in HDS of straight-run gas oil [5,6,7].

An alternative to enhance the performance in HDS reactions is the use of organic additives [8,9]. The organic additives have been demonstrated to increase the catalytic activity on HDS reactions such as thiophene, dibenzothiophene and 4,6-dibenzotiophene [10,11,12,13]. The most common organic additives used in the synthesis of CoMo, NiMo and NiW catalysts are citric acid [11,13,14], ethylenediaminetetraacetic acid (EDTA) [8,12,15] or *trans*-1,2-cyclohexanediamine-*N*,*N*,*N*′,*N*′-tetraacetic acid (CyDTA) [10,16,17]. In these studies, the chelating agents form metal-organic species improved the metal-support interaction, MoS_2_ (WS_2_) dispersion, and sulfidation degree of Mo and Co species. In addition, the organic-metal species avoid the migration of the particles such as Co or Ni, and therefore, favor the promotion of Co(Ni) to form the most active species in HDS reaction “CoMoS (NiWS or NiMoS)”.

On the other hand, the role of the organic additive in the preparation stage takes relevance on the synthesis of HDS catalysts [18]. Some authors have demonstrated that the organic additive improves the formation of metal-organic species when co-impregnating the metals and the organic additives [10,11]. Moreover, the increment of the organic-metal species favored the formation of the active NiWS species. However, other authors suggest that the addition of organic agents in sequential impregnations leads to a better metal–support interaction, which enhances the dispersion of the MoS_2_ (WS_2_) [13,19].

Based on the above information, the use of the organic additive is an interesting topic in order to improve the activity of HDS catalysts. In this aspect, 2,6-bis-(1-hydroxy-1,1-diphenyl-methyl) pyridine (BDPHP) showed the ability to form penta coordinated complexes with Sn [20]. Moreover, the use of BDPHP as organic additive for the preparation of CoMo, NiMo or NiW HDS catalysts has not been reported. Therefore, the aim of this work was to study the Ni-Mo dispersion and the interaction with BDPHP, where the different BDPHP/Ni molar ratios were synthesized to elucidate the effect of the concentration of BDPHP on the catalytic surface with the HDS of straight-run gas oil.

## 2. Experimental

### 2.1. Catalyst Preparation

The γ-Al_2_O_3_ was modified by surface impregnation of a solution of BDPHP to afford BDPHP/Ni molar ratios (0.5 and 1.0) in the final composition. The dried support with BDPHP was co-impregnated using a solution of molybdenum oxide [MoO_3_], nickel carbonate hydroxide tetrahydrate [(2NiCO_3_·3Ni(OH)_2_·4H_2_O)] and phosphoric acid [H_3_PO_4_]. For all catalysts, the nominal metal contents were 12.0, 3.0 and 1.6 wt % for Mo, Ni and P, respectively. The calcination was avoided to evade the decomposition of the BDPHP. The catalysts are labeled NiMoP(x), where x is the molar ratio BDPHP/Ni at 0.5 and 1.0. Sample NiMoP/γ-Al_2_O_3_ was used as reference and it was prepared without organic additives and named NiMoP (0.0).

### 2.2. Characterization Techniques

Textural properties of various materials were determined by N_2_ physisorption (−196 °C), in an Autsorb-1 (Quantachrome, Boynton Beach, FL, USA) apparatus. Surface area and pore size distribution of prepared solids were determined by Brunauer-Emmett-Teller (BET) and Barret-Joyner-Halenda (BJH) (N_2_ adsorption branch data) protocols, respectively. The crystalline components of the materials were examined by X-ray diffraction (XRD) using Siemens D-500 kristalloflex (Munich, Germany), Cukα radiation, λ = 0.15406 nm, 35 kV, 25 mA. Phase identification based on XRD patterns was supported by the ICDD-PDF-2-database. The Fourier transform infrared spectra of the solids were recorded in the transmittance mode on a Perkin Elmer Spectrum One (Waltham, MA, USA) with a universal attenuated total reflectance (ATR) sampling accessory in the 1800–1300 cm^−1^ range. Spectra were measured at room temperature at a 4 cm^−1^ resolution and an average of 256 scans per sample was used. Raman spectra were obtained at room temperature on a T64000 triple monochromator (Jobin-Yvon-Horiba, Edison, NJ, USA) using the 514.5 nm line of an Ar+ laser (Lexel Laser, Fremont, CA, USA). All the spectra were obtained at a power of 10 mW at the laser head, in the range 10–1600 cm^−1^, using an Olympus microscope (Shinjuku, Tokio, Japan) with a 100× objective and 10 accumulations of 60 s each. The spectrum resolution was 1 cm^−1^.

Thermal analyses (from room temperature to 1000 °C) of freshly dried samples (ca. 15 mg) were carried out with a Netzch Thermische Analize, STA 409 EP apparatus (Selb, Germany) under a static air atmosphere, operating at 10 °C/min heating ramp. Temperature programmed reduction (TPR) of the catalysts were performed using Altamira Instruments AMI-90 equipped with a thermal conductivity detector (TCD). Approximately 0.1 g of catalyst sample was placed in a quartz sample cell (U-shaped) and then pretreated in situ at 393 K for 1 h under Ar flow. The reduction of catalysts was performed from room temperature to 1273 K, under a stream of 10% H_2_, employing a gas flow rate of 0.84 cm^3^/s and heating rate of 10 °C/min.

XPS spectra of the sulfided catalyst samples were measured at room temperature using a VG Escalab 200R spectrometer (Richardson, TX, USA) equipped with a hemispherical electron analyzer and a MG Kα (hν = 1253.6 eV) X-ray source. The details of the XPS measurements by this spectrometer are reported elsewhere [21].

### 2.3. Catalytic Activity

The solid catalysts (1.20 mL; 80–100 mesh) were sulfided in virgin straight run gas oil (2.05 wt % S) spiked with dimethyl disulfide (DMDS) to afford 2.5 wt % of sulfur in the feedstock and then fed to the reactor at a flow rate according to an liquid hourly space velocity (LHSV) of 2.5 h^−1^. The pressure was set to 56 kg/cm^2^ and the hydrogen rate was adjusted according to a H_2_/oil ratio of 500 m^3^ (STP)/m^3^. The temperature of the catalyst bed was increased to 290 °C and held for 15 h. These were tested in the HDS reaction of SRGO and H_2_ flow rates were adjusted to LHSV 1.5 h^−1^, 370 °C, H_2_/oil ratio equal to 500 m^3^ (STP)/m^3^ and 56 kg/cm^2^. The sulfur concentration in liquids was obtained with a Tanaka RX-360SH model sulfur analyzer according to the ASTM D4294 method. For each test, 10 mL of sample was fed to the equipment. The reported instrument sensitivity is ±0.001% of the measured value.

The apparent rate constant rates (k_hds_(Sw%)^−0.5^ h^−1^) were estimated at steady state conditions (after 12 h), and they were calculated according to the following equation [22,23]:

(1) $$k_{hds}=\frac{LHSV}{n-1}\left(\frac{1}{S_{p}^{n-1}}-\frac{1}{S_{f}^{n-1}}\right),$$

where:

*k_hds_*: Pseudo 1.5 order HDS kinetic constant (Sw%^−0.5^ h^−1^);
*S_p_*: Sulfur in product (wt %);
*S_f_*: Sulfur in feedstock (wt %);
*LHSV*: Liquid Hourly Space Velocity (h^−1^).

## 3. Results

### 3.1. N_2_ Physisorption (N_2_-Phys)

Table 1 shows the textural properties for NiMoP (0.0), NiMoP (0.5) and NiMoP (1.0). The reference catalyst showed lower specific area, pore volume and pore size in comparison with the catalysts with the organic additive. The NiMoP (1.0) presented similar pore size and total volume to NiMoP (0.5). However, the specific area for NiMoP (0.5) slightly decreased (156 vs. 164 m^2^/g) in contrast with NiMoP (1.0). In addition, the catalysts with organic additive have more specific area than the NiMoP (0.0) suggesting that the BDPHP slightly modifies the textural properties.

### 3.2. X-ray Diffraction (XRD)

**Figure 1:**
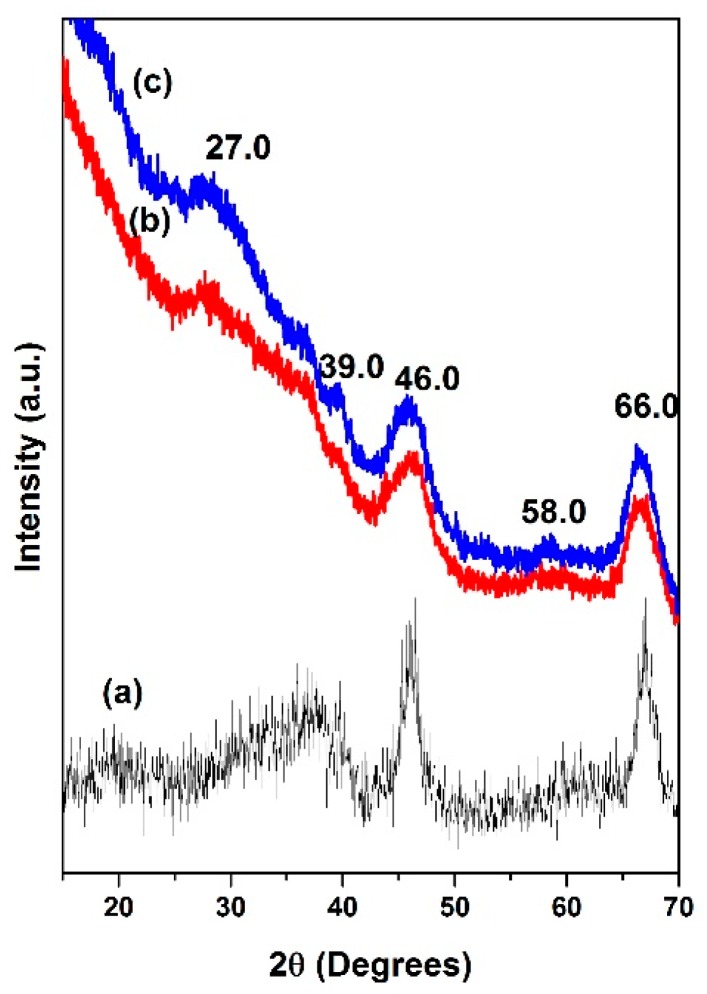
X-ray diffraction for NiMoP/γ-Al_2_O_3_ at different molar ratios of BDPHP/Ni: γ-Al_2_O_3_ (a); 0.5 (b); and 1.0 (c).

The X-ray diffractions pattern shown in Figure 1 reveals that the catalysts displayed peaks related to different crystal structure. The NiMoP/γ-Al_2_O_3_ at different molar ratios of BDPHP/Ni showed peaks at 66° and 46° corresponding to 400 and 440 planes, respectively, from the γ-Al_2_O_3_ pattern (Figure 1) [24,25]. The NiMoP (0.5) and NiMoP (1.0) catalysts exhibited a peak at 27° that can be associated with 020 planes of MoO_3_ [26]. In addition, NiMoP (1.0) presented peaks at 39° (030 planes) and 58 °C, which is related to MoO_3_, while the NiMoP (0.5) exhibited only a peak at 58° [26].

In contrast, the NiMoP (0.5) did not show the peak at 39° corresponding to MoO_3_ in comparison with NiMoP (1.0). This result suggests that the NiMoP (0.5) have more MoOx species with a lower size than the NiMoP (1.0). Therefore, NiMoP (0.5) has a higher dispersion of MoO_3_ than NiMoP (1.0) [27].

### 3.3. Fourier Transformed Infrared Spectroscopy (FT-IR)

**Figure 2:**
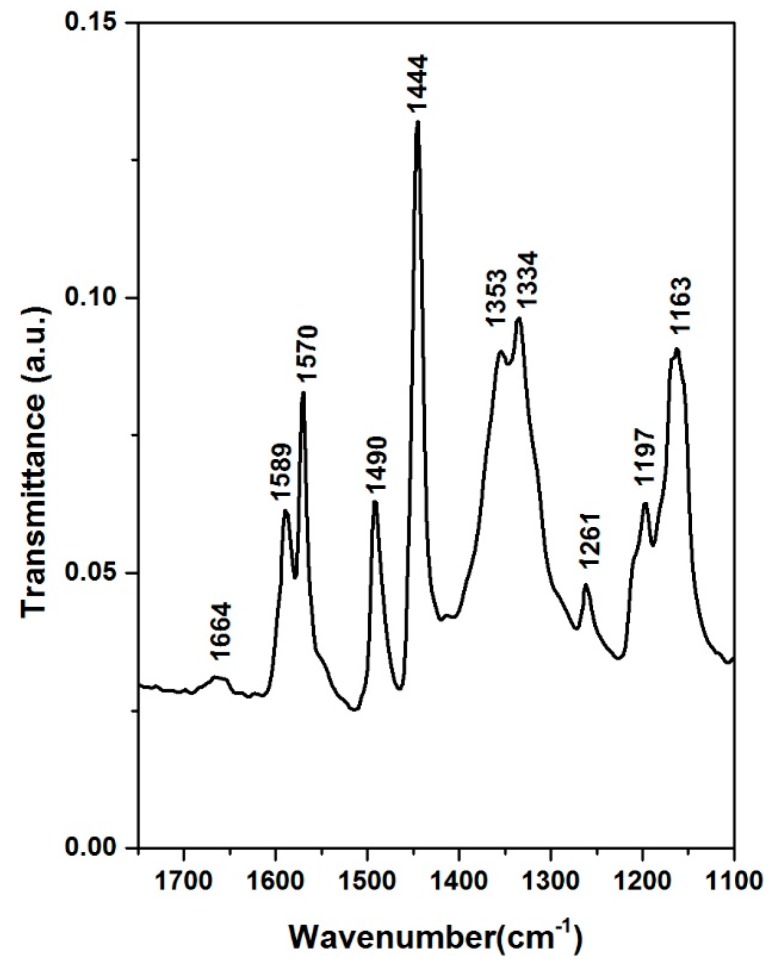
Fourier transform infrared spectra for 2,6-Bis-(1-hydroxy-1,1-diphenyl-methyl) pyridine.

**Figure 3:**
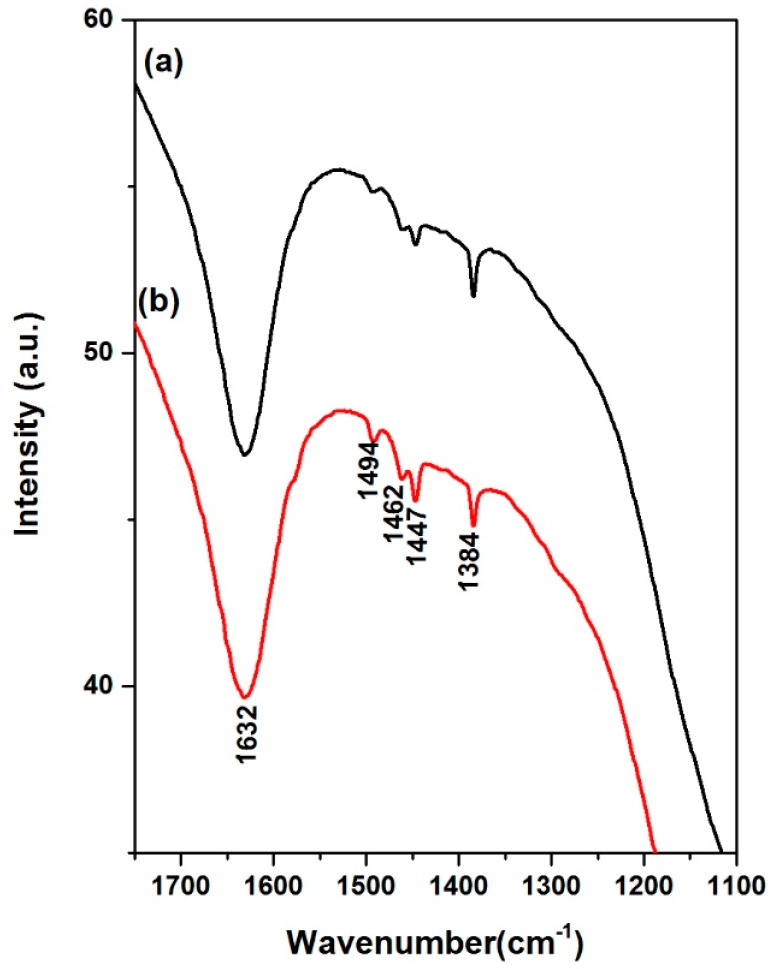
Fourier transform infrared spectra for NiMoP/γ-Al_2_O_3_ at different molar ratios of BDPHP/Ni: 0.5 (a) and 1.0 (b).

The FT-IR spectroscopy allowed us to identify the vibrations of BDPHP on the catalyst NiMoP/γ-Al_2_O_3_ before the HDS reaction. Figure 2 and Figure 3 show the spectra for 2,6-Bis-(1-hydroxy-1,1-diphenyl-methyl) pyridine and NiMoP/γ-Al_2_O_3_ at different BDPHP/Ni molar ratio (0.5 and 1.0). The peaks in the 1800–1100 cm^−1^ frequency region are attributed to the carbon and nitrogen vibrations [28,29,30]. In this region, the spectra of BDPHP exhibited some vibrational frequencies corresponding to the ring vibration (1664 and 1589 cm^−1^), C=C (1570 cm^−1^), C=N (1490 cm^−1^), C-C (1444 and 1353 cm^−1^), C-NH_2_ (1334 cm^−1^), C-N (1261 cm^−1^) and C-H (1197 and 1163 cm^−1^) [28,29,30].

The NiMoP (0.5) and NiMoP (1.0) exhibited vibrational frequencies corresponding to C-C vibration at 1462, 1447 and 1384 cm^−1^ [28,29,30]. Moreover, the NiMoP (0.5) and NiMoP (1.0) exhibited a peak at 1632 cm^−1^ corresponding to Al-O-C vibration [29,30,31]. Furthermore, the CoMoP (0.5) and CoMoP (1.0) presented C=N vibration at 1494 cm^−1^ [28,29,30]. In contrast with BDPHP spectra (Figure 2), the catalysts presented the C=N vibration close to the wavenumber of BDPHP. This result discards the formation of Ni or Mo interaction with the free pair of electrons of C=N from the organic additive. In addition, this result is related to the low ability complex for some organic additives with a larger size and some metals [32]. However, the OH groups of the organic additive contribute to the formation of new Al-O-C [31]. In this line, the BDPHP covered the γ-Al_2_O_3_ prevented the strong metal-support interaction [31,33].

### 3.4. Raman Spectroscopy

**Figure 4:**
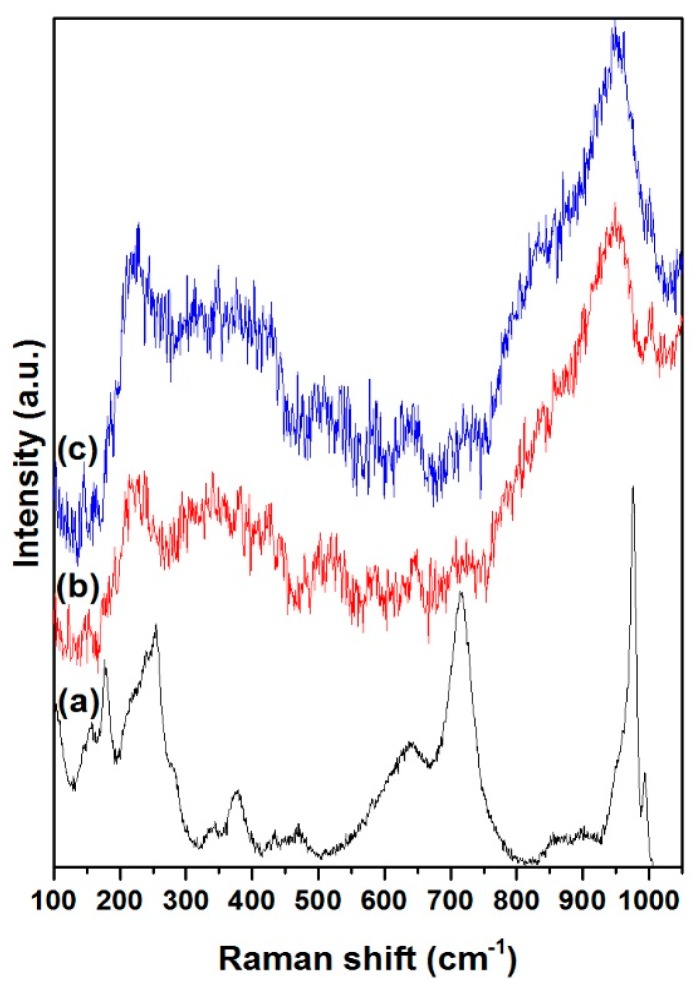
Raman spectra for NiMoP/γ-Al_2_O_3_ at different molar ratios of BDPHP/Ni: 0.0 (a); 0.5 (b); and 1.0 (c).

Raman spectroscopy was used to determine the Mo species in NiMoP/γ-Al_2_O_3_ catalysts at different molar ratios of BDPHP/Ni. Figure 4 shows the Raman spectra for NiMoP (0.0), NiMoP (0.5) and NiMoP (1.0), which is supported by γ-Al_2_O_3_. The NiMoP (0.0) spectrum exhibiting bands at 176, 255, 376, 638, 714, 853, 975, and 993 cm^−1^. The bands at 993, 975, 376, 225, 376 and 176 cm^−1^ are assigned to Mo_7_O_24_^6−^ [34], while the intense 853 cm^−1^ signal is ascribed to large MoO_3_ aggregates [35]. The peaks at 638 and 714 cm^−1^ are associated with the γ-Al_2_O_3_ [36]. The NiMoP with BDPHP/Ni (0.5, 1.0) spectra showed bands at 220, 360, 565, 895, 950 and 1015 cm^−1^. The band at 895 cm^−1^ corresponds to MoO vibration of MoO_4_^2−^ while the bands at 220, 360, 565 and 950 cm^−1^ are associated with Mo_7_O_24_^6−^ species [34]. Additionally, the peak at 1015 cm^−1^ is associated with P-O vibrations [28,34]. Regarding the band located at 950 cm^−1^, it increases gradually conversely with the decrease of the BDPHP/Ni ratio, most probably due to the BDPHP, which promotes the polymerization of the polymeric Mo_7_O_24_^6−^ species. This, in fact, suggests changes in the Mo oxide dispersion with the addition of BDPHP.

**Figure 5:**
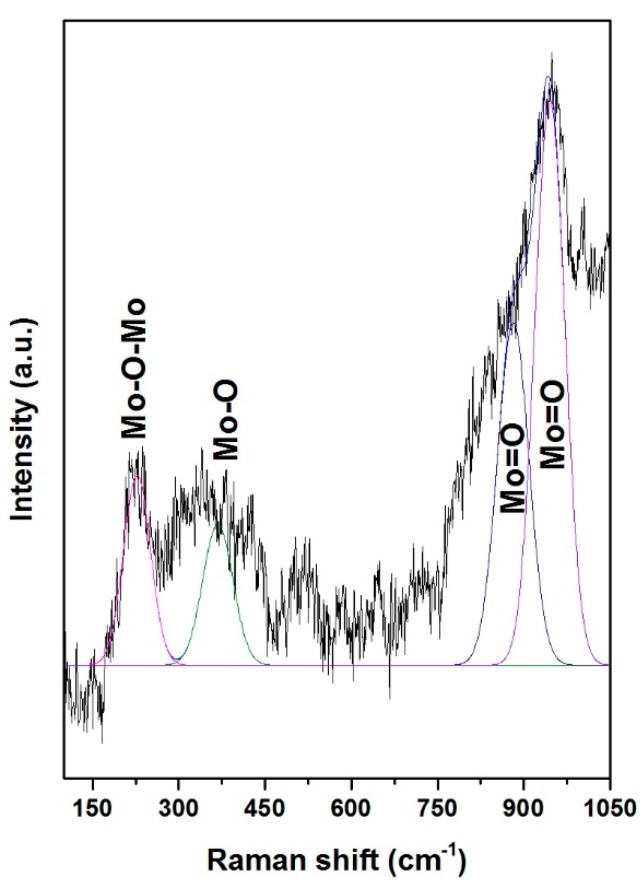
Gaussian deconvolution of NiMoP/γ-Al_2_O_3_ at BDPHP/Ni = 0.5.

**Figure 6:**
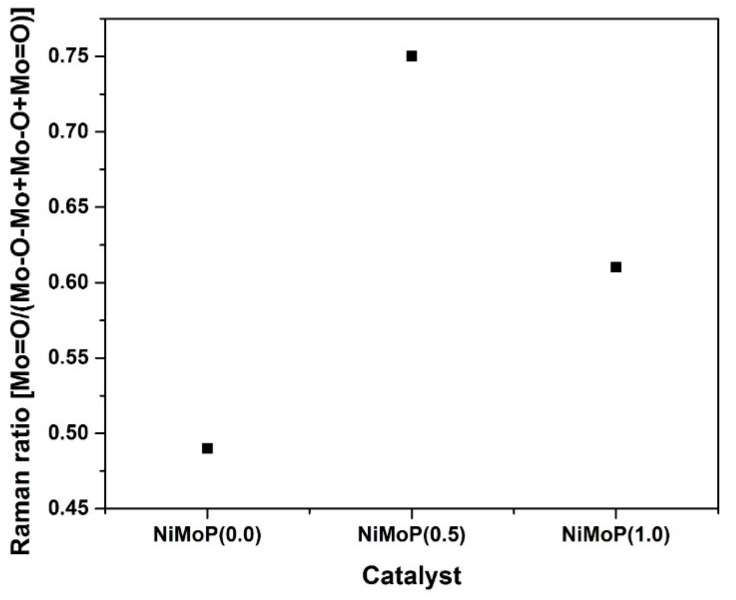
Raman Ratio as a function of the molar ratio BDPHP/Ni in the NiMoP catalysts.

The Mo species at 220 and 360 cm^−1^ is associated with the Mo-O-Mo [37]. In addition, the Mo species at 895 and 950 cm^−1^ is ascribed to the Mo=O vibrations [37]. To obtain information about the Mo oxide dispersion, the Raman spectra were deconvoluted with the Mo=O, Mo-O and Mo-O-Mo vibrations. Figure 5 shows the deconvolution for NiMoP (0.5). Figure 6 exhibits the Raman ratio Mo=O/(Mo=O + Mo-O + Mo-O-Mo) of NiMoP catalysts at different BDPHP/Ni ratios. Figure 6 showed that the Raman ratio increases until a maximum at BDPHP/Ni = 0.5, and then decreases at BDPHP/Ni = 1.0. This result suggests the re-dispersion of Mo oxide provoked by BDPHP with different BDPHP/Ni molar ratio. A similar behavior was seen by Diaz de Leon [38] in an analogous system with additives. Regarding this, Diaz de Leon suggested that the increase of the Raman band intensities ratio promoted better dispersion of the metal species and is responsible for the highest HDS activity.

### 3.5. Thermal Analysis (TGA/DTA)

**Figure 7:**
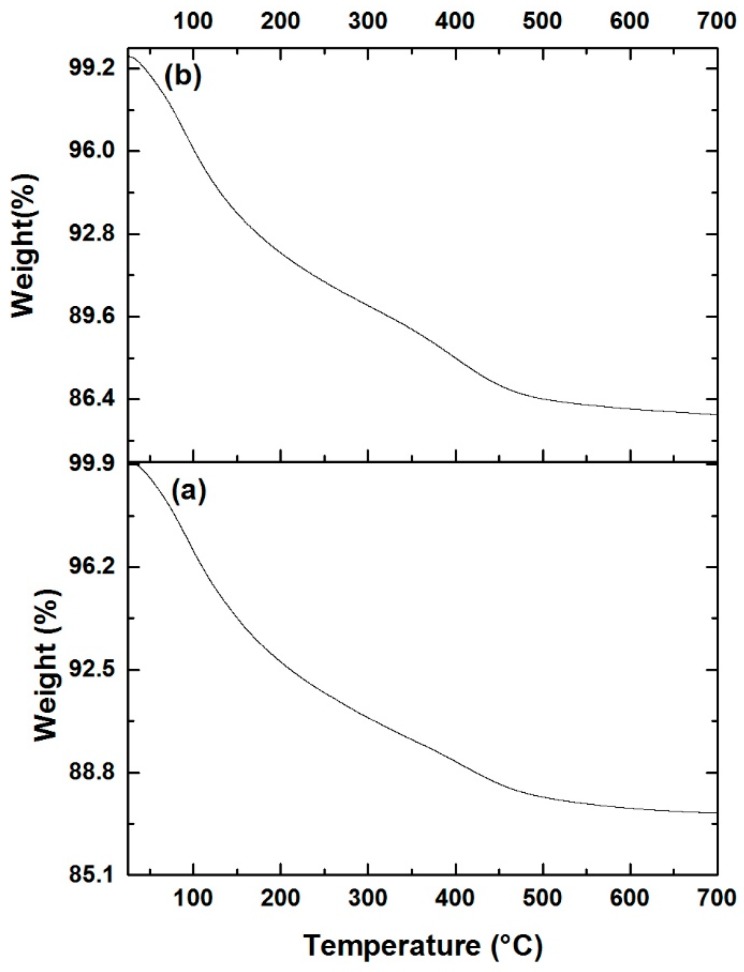
Thermogravimetric analysis for NiMoP/γ-Al_2_O_3_ at different molar ratios of BDPHP/Ni: 0.5 (**a**) and 1.0 (**b**).

**Figure 8:**
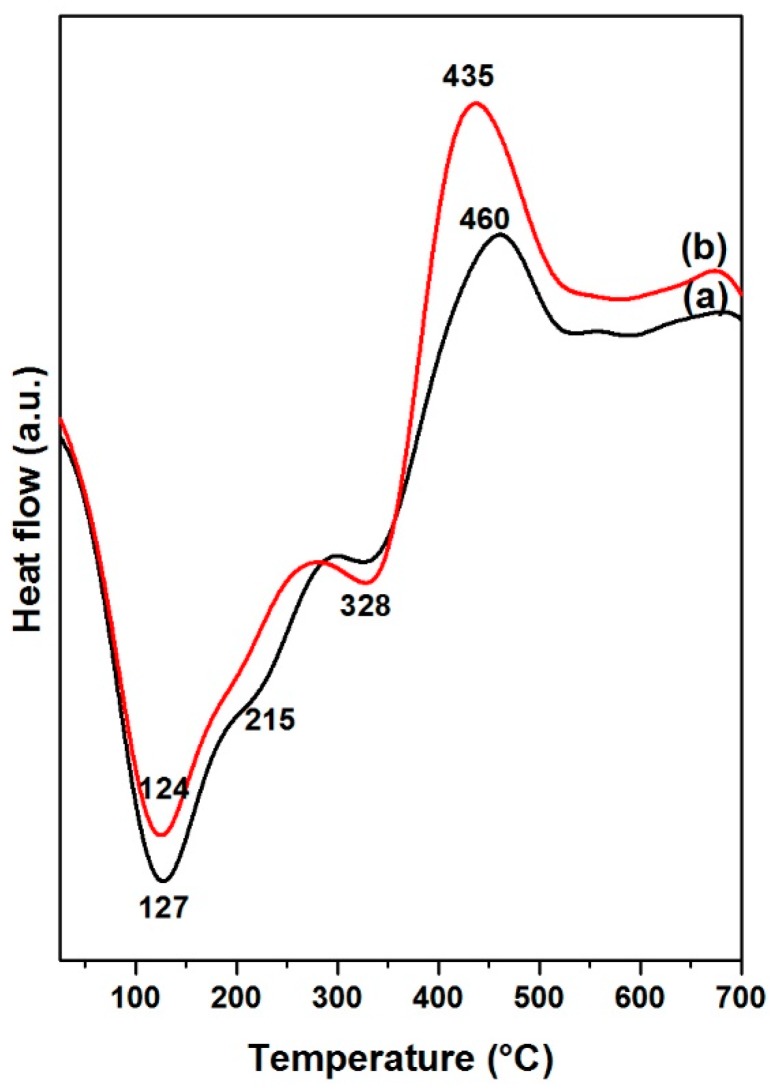
Heat flow profiles for NiMoP/γ-Al_2_O_3_ at different molar ratios of BDPHP/Ni: 0.5 (a) and 1.0 (b).

To study the thermal stability of the catalysts with BDPHP, TGA analysis of NiMoP (0.5) and NiMoP (1.0) samples were carried out and the results are shown in Figure 7 and Figure 8. It can be seen that the TGA curves show a continuous weight loss of 87.3 and 85.8 wt % from 30 to 700 °C for CoMoP (0.5) and CoMoP (1.0), respectively. The weight loss for all catalysts around 12.0 wt % lower than 200 °C is associated with the removal of physisorbed H_2_O [39], and the further weight loss around 75.0 wt % is attributed to the loss of crystallization H_2_O and the stepwise loss of the organic additive [40].

The heat flow profiles for NiMoP (0.5) and NiMoP (1.0) samples (Figure 8) exhibited a broad endothermic peak of around 100–200 °C related with dehydration reactions [39]. The exothermic peaks centered in the 430–460 °C range on the heat flow profiles are associated with the decomposition of BDPHP (decarboxylation reactions) for NiMoP (0.5) and NiMoP (1.0) samples [40]. The exothermic peak maximum decreases in the order of NiMoP (0.5) > NiMoP (1.0). It is supposed that a higher decomposition temperature corresponds to major interaction between the support and the organic additive. In contrast, it is suggested that the NiMoP (0.5) catalyst presented higher interaction.

### 3.6. Temperature Programmed Reduction (TPR)

**Figure 9:**
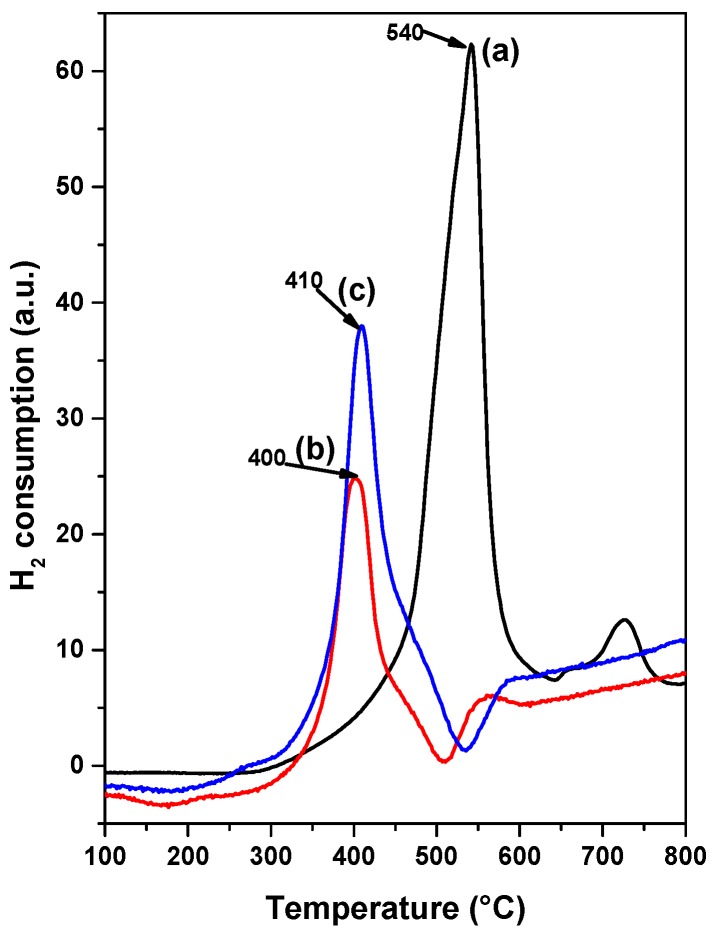
Temperature-programmed reduction profiles for NiMoP/γ-Al_2_O_3_ at different molar ratios of BDPHP/Ni: 0.0 (a); 0.5 (b); and 1.0 (c).

The influence of the organic additive on the nickel-molybdenum-support interaction was investigated by temperature-programmed reduction of NiMoP/γ-Al_2_O_3_. The TPR profiles of NiMoP (0.0), NiMoP (0.5) and NiMoP (1.0) are shown in Figure 9. From Figure 9, the NiMoP (0.0) sample displayed two reduction peaks at 540 °C and 725 °C, which is associated with the nickel-molybdenum and the molybdenum reduction, respectively. NiMoP (0.5) presents two reduction peaks at 400 °C and 555 °C, corresponding to the decomposition/reduction of nickel-molybdenum and molybdenum precursor in weak and strong interactions with the γ-Al_2_O_3_ support, respectively [41,42,43,44]. Additionally, a shoulder can be observed at 222 °C, which corresponds to the reduction of nickel in a weak interaction with γ-Al_2_O_3_ [43]. The reduction profiles of NiMoP (1.0) exhibit two peaks: one at 410 °C ascribed to the reduction of nickel-molybdenum species, and the second one at about 586 °C due to the reduction of molybdenum species [41,42,43,44]. This catalyst also displays a peak at 271 °C corresponding to the nickel reduction [43].

The increase in the Ni and Mo reduction temperature due to the presence of higher concentration of BDPHP on the NiMoP (1.0) catalyst suggests the interaction between BDPHP and Mo-Ni species in NiMoP/γ-Al_2_O_3_. This effect was observed previously for an analogous system synthesized in the presence of EDTA [8]. Moreover, the lower temperature reduction of Ni and Mo species for NiMoP (0.5) can be rationalized in terms of better interaction of metal-support in comparison with the NiMoP (1.0) and NiMoP (0.0), and, likewise, the easier reduction of the NiMoP (0.5), compared with the NiMoP (0.0) sample, which is attributed to the presence of the organic additive at lower concentration favoring the nucleation step. In this line, the easier reduction improves the sulfidation degree of Mo and Ni species [45].

### 3.7. X-ray Photoelectron Spectroscopy of Sulfided NiMoP/γ-Al_2_O_3_ Catalyst (XPS)

**Figure 10:**
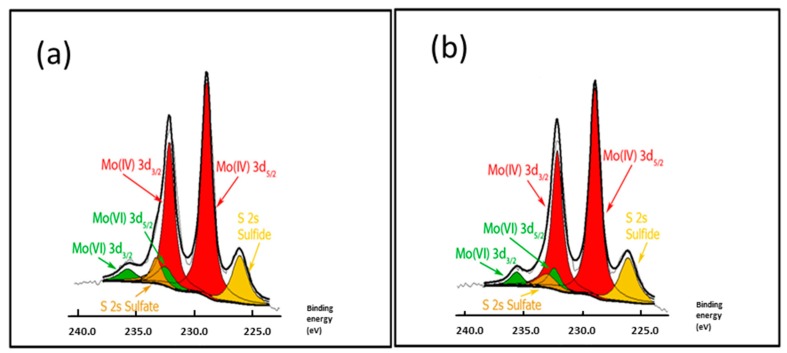
X-ray photoelectron spectra of sulfided NiMoP/γ-Al_2_O_3_ catalysts: For the NiMoP sample, deconvolution in Mo 3d emission line is shown in the inset of this figure: NiMoP (0.5) (**a**) and NiMoP (1.0) (**b**).

To monitor the evolution of the sulfide surface species, XPS was studied in the Mo 3d and Ni 2p levels. As an example, the XPS spectra of Mo 3d emission line and fit decomposition of NiMoP (0.5) and NiMoP (1.0) samples are presented in Figure 10. The Mo 3d core-level spectra of all catalysts were found to be rather complex, suggesting the presence of at least two species. They are fitted satisfactorily with two sets of doublets, each one containing the Mo 3d_5/2_ and Mo 3d_3/2_ components coming from the spin-orbit splitting. The observation of two doublets indicates that there are two different Mo-species. The Mo 3d_5/2_ peak for the first set, at 228.92 eV, is assigned to MoS_2_ (Mo^4+^) species, and the other at 232.88 eV is attributed to Mo^6+^ species [46,47,48,49] (Table 2). The NiMoP reference catalyst without organic additive exhibited 70% of MoS_2_ species. For the NiMoP (0.5) catalyst, 86% of Mo species was converted to MoS_2_ species while the NiMoP (1.0) showed 87%. The XPS results suggest that the organic additive increases the sulfidation degree in the NiMoP/γ-Al_2_O_3_ system.

**Figure 11:**
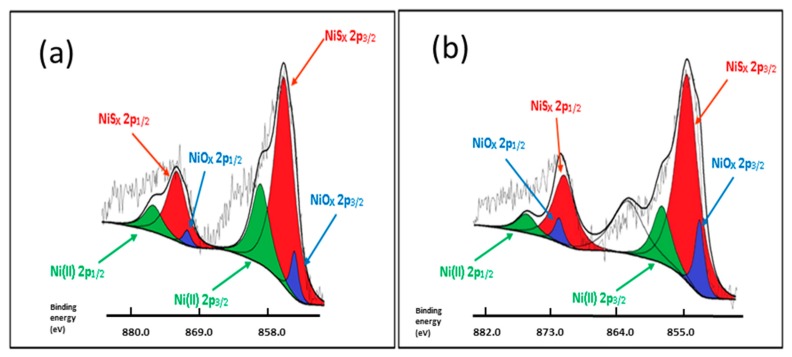
X-ray photoelectron spectra of sulfided NiMoP/γ-Al_2_O_3_ catalysts: For the NiMoP sample, deconvolution in Ni 2p emission line is shown in the inset of this figure: NiMoP (0.5) (**a**) and NiMoP (1.0) (**b**).

The XPS spectra of the Ni 2p emission line for all fresh sulfided catalysts are presented in Figure 11. As can be seen from this figure, spectra show primary satellite peaks due to shake-up electrons. Since the line-shape of the Ni 2p_3/2_ peak, and especially its satellite, was unsymmetrical and broad, we applied an empirical method to roughly fit the curve using Gaussian/Lorentzian distributions. An example of the decomposition of Ni 2p_3/2_ profile made for the NiMoP sample is shown in the inlet of Figure 11. Each Ni 2p_3/2_ profile was resolved in three components at 863.8, 857.0 and 860.5 eV, corresponding to highly dispersed NiS, NiMoS and NiOx phases, respectively [46,47,48,49]. Apparently, the electronegativity of the organic additive has little effect on the binding energies separating satellite and main peaks of the Ni 2p_3/2_ spectra. The BE at 853.1 eV is close to the value reported in the literature for bulk NiS on unsupported MoS_2_ (852.9 eV) [38], whereas the BE at 854.4 eV is close to the value reported for NiMoS species formed after sulfidation of the NiMo/γ-Al_2_O_3_ catalysts at 673 K [46,47,48,49].

Considering the binding energy position and the relative area related to the three Ni species, the percentages of each species were determined. As seen in Table 2, the reference catalyst displayed 49% of NiMoS species [31]. For the catalysts with organic additives, the NiMoP (0.5) sample exhibited 45% of the NiMoS species, while the NiMoP (1.0) with a higher concentration of BDPHP showed increased percentages of this species to 46%. The Ni and Mo species surface exposure and the catalyst sulfidation degree could be deduced from Table 3 showing (Ni + Mo)/Al and S/(Ni + Mo) atomic ratios, respectively. The samples prepared with BDPHP/Ni = 0.5 exhibit a lower Mo and Ni species surface exposure than the BDPHP/Ni = 1.0, as inferred from the comparison of the (Ni + W)/Al atomic ratio (Table 3). However, the catalysts with BDPHP showed more metal species over the surface in comparison with the reference catalyst. Considering the TPR results (vide supra), this might indicate that catalyst sulfidation at 290 °C led to the migration of the metal species from the support surface to its inner porous structure. Finally, there is a significant variation in the sulfidation degree of the Mo species, as deduced from the comparison of the S/(Mo + Ni) atomic ratios (Table 3). The NiMoP (0.5) exhibited 30% more sulfidation degree than NiMoP (1.0) and NiMoP (0.0).

### 3.8. Fourier Transformed Infrared Spectroscopy of Sulfided NiMoP/γ-Al_2_O_3_ Catalysts

**Figure 12:**
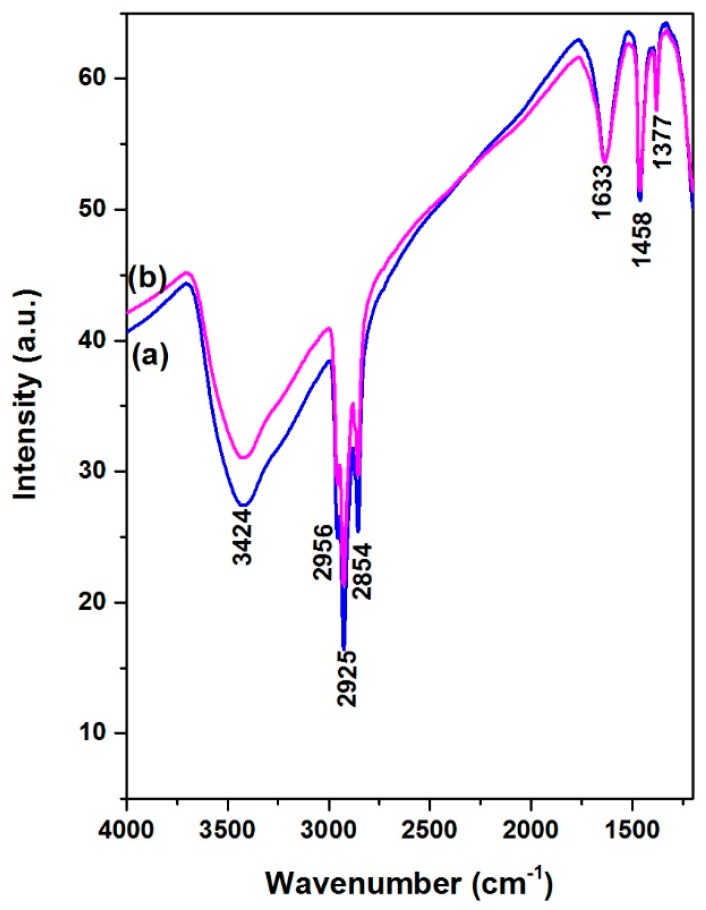
FT-IR spectra for NiMoP/γ-Al_2_O_3_ after reaction at different molar ratios of BDPHP/Ni: 0.5 (a) and 1.0 (b).

The FT-IR spectra for NiMoP/γ-Al_2_O_3_ after reaction at different molar ratios of BDPHP/Ni (0.5, 1.0) given in Figure 12. The NiMoP/γ-Al_2_O_3_ (0.5) and NiMoP/γ-Al_2_O_3_ (1.0) exhibited peaks at 3424, 2956, 2925 and 2854 cm^−1^ corresponding to CH_3_, CH_2_ and CH vibrations [50,51,52]. These vibrations are related to coke formation from the gas oil after the reaction. The catalysts also showed peaks at 1633, 1458 and 1377 cm^−1^ corresponding to coke formation after HDS of gas oil [50,51,52]. In addition, the coke vibration presented lower intensity for the NiMoP (0.5) in contrast with the NiMoP (1.0). This augment of coke affects the MoS_2_ species for HDS reaction, which is in agreement with the XPS results.

### 3.9. Catalytic Activity Measurements

The activity of the sulfided catalysts was evaluated in the HDS of straight-run gas oil and the reaction was carried out in a fixed bed reactor (T = 340 °C and 56 kg/cm^2^ of pressure). A conventional NiMoP/γ-Al_2_O_3_ catalyst with similar metal content (12.0, 3.0 and 1.6 wt % for Mo, Ni and P, respectively) was used as reference. The straight-run gas oil conversion and the reaction rate constants are listed in Table 4. The catalyst prepared with BDPHP at lower concentration (BDPHP/Ni = 0.5) exhibits a higher straight-run gas oil conversion than the NiMoP (0.0) and NiMoP (1.0). Thus, to clarify the effect of organic additive, the reaction rate constants (at steady state) should be compared with their Raman ratios where the HDS activity and the Raman ratio exhibits a relationship (Table 4). The most active catalyst NiMoP (0.5) exhibits the highest Raman ratio value. When BDPHP/Ni ratio increased at 1.0, the activity decreased. At the same time, the lowering of the Raman ratio occurs. Interestingly, the reaction rate constant of NiMoP (0.5) increased 40% compared with the NiMoP (0.0) and NiMoP (1.0) samples. It is clear that the variations in the catalytic activity are related to the presence of the Mo=O species. However, BDPHP/Ni ratio conditions should be controlled, since the best activity was achieved for the sample prepared at BDPHP/Ni = 0.5.

## 4. Discussion

The NiMoP (0.5) catalyst was more active in the HDS of straight-run gas oil than NiMoP (1.0) and NiMoP (0.0). It was demonstrated that the final characteristic of the dried and sulfided NiMoP/γ-Al_2_O_3_ samples depends strongly on the BDPHP/Ni molar ratio; with 0.5 being the optimal molar ratio. At this ratio, there is a chemical equilibrium between the BDPHP located in the interface region and -OH groups of the alumina support. The role of the interface region on the adsorption of an organic additive such as BDPHP is extensively discussed in the review by Bourikas et al. [53,54]; therefore, it will be not discussed in this work.

The N_2_-phys study showed that the NiMoP (1.0) presented more surface area than the NiMoP (0.5) and NiMoP (0.0). On the other hand, XRD showed peaks corresponding to Mo species. In addition, NiMoP (1.0) exhibited two additional peaks at 39 °C and 58 °C corresponding to Mo species, indicating that the NiMoP (1.0) presented more Mo species with higher size in comparison with NiMoP (0.5), which has more dispersion than NiMoP (1.0).

The FT-IR results displayed that the BDPHP did not interact with Mo or Ni species. However, we do not discard an interaction between BDPHP and superficial groups from γ-Al_2_O_3_. Furthermore, the increment of the coke signal was seen for NiMoP (1.0). In this sense, the coke affects the MoS_2_ species for HDS reaction. On the other hand, the Raman spectroscopy suggests an increment of the amount of superficial Mo=O for NiMoP (0.5) than NiMoP (1.0) and NiMoP (0.0). This result suggests that the NiMoP (0.5) displays higher oxide dispersion than its NiMoP (0.0) counterpart. This finding is in agreement with the XRD and XPS results. Likewise, this increment of MoS_2_ dispersion seen by XPS is related to the isolating effect proposed by Li [19]. Accordingly, during the sulfurization process (exothermic reaction [55]), the carbonaceous deposits may isolate the Mo species from the γ-Al_2_O_3_ and favored the formation of MoS_2_ on the surface.

The TPR profiles showed that the temperature reduction of Ni-Mo species for NiMoP (0.5) shifted to low temperature in contrast with NiMoP (1.0) and NiMoP (0.0). These results suggest a better interaction of metal–support and easier reduction for NiMoP (0.5). This fact could suggest that the addition of BDPHP has an effect on the Ni and Mo sulfidation. In this line, the XPS results displayed an increment of 30% in the sulfidation degree for NiMoP (0.5), in comparison with NiMoP (1.0) and NiMoP (0.0). Consequently, an increment of the Mo sulfidation improves the active phase formation (MoS_2_). Moreover, the content of coke was higher for NiMoP (1.0) in comparison with NiMoP (0.5). This result is in accordance with FT-IR measurement for NiMoP after HDS reaction.

**Figure 13:**
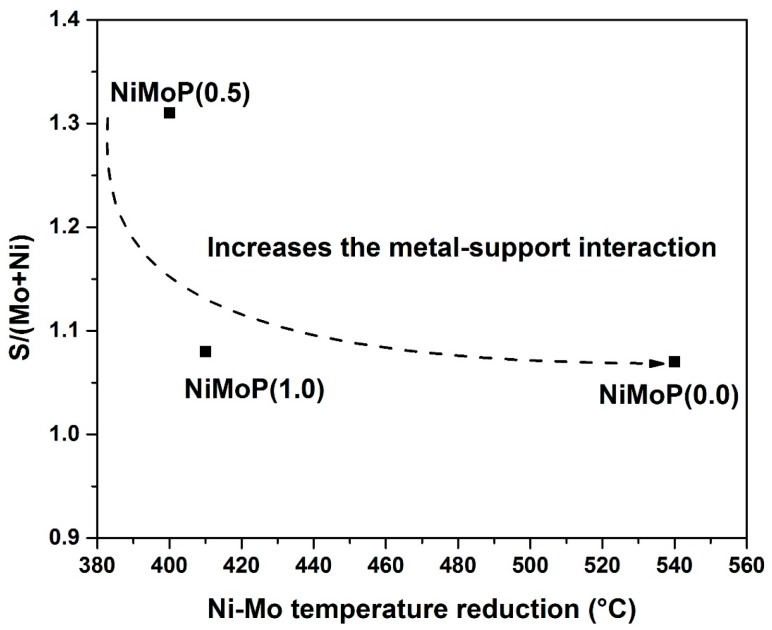
Relation between Ni-Mo temperature reduction with sulfurization degree of Ni and Mo.

**Figure 14:**
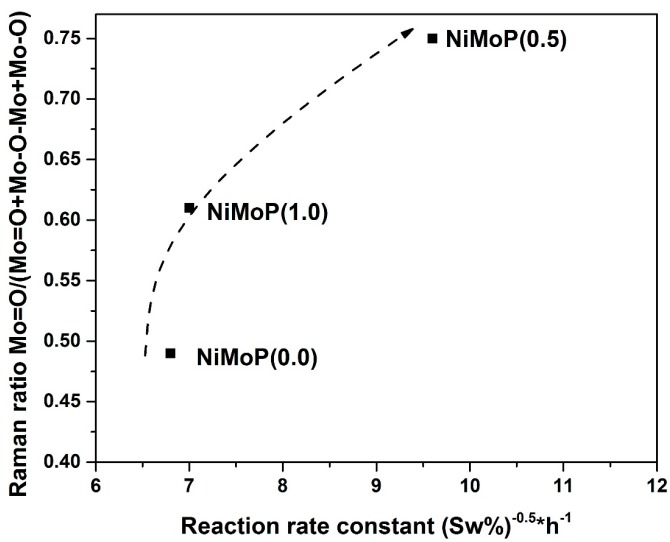
Relation between Raman ratio and reaction rate constant for NiMoP/γ-Al_2_O_3_ at different molar ratios of BDPHP/Ni: 0.0, 0.5 and 1.0.

From the TPR and XPS characterization, a relation was found between the S/(Mo + Ni) and Ni-Mo temperature reduction for NiMoP (0.0), NiMoP (0.5) and NiMoP0 (1.0) catalysts (Figure 13) in addition to the reaction rate constant and Raman ratio (Figure 14). The sulfidation degree increases with a decrease in the temperature reduction of Ni and Mo species. This effect is related to a decrease of the metal-support interaction [45]. On the other hand, the catalytic activity increases with the MoOx species as Mo=O. These results are in agreement with the fact that Mo=O species are more available for sulfidation process than the Mo-O and Mo-O-Mo species [38]. The latter species are close to the support; therefore, these are difficult to sulfide. Additionally, the highest activity of the NiMoP (0.5) catalyst with respect to NiMoP (1.0) and NiMoP (0.0) samples are linked to its moderate metal–support interaction, higher sulfidation degree, lower content of coke, and higher amount of Mo=O species.

## 5. Conclusions

In summary, a significant increase in the HDS activity of NiMoP/γ-Al_2_O_3_ catalysts was found when the support was impregnated with BDPHP organic additive at BDPHP/Ni = 0.5. The addition of BDPHP covered the Al_2_O_3_ surface in order to isolate the metals and the support. The increase of HDS activity of the catalysts with organic additive was associated with the formation of Mo=O species, enhancement of the MoO_3_ dispersion, moderate metal-support interaction and an increment of Ni and Mo sulfurization degree.

## Figures and Tables

**Table 1:** Textural properties for NiMoP/γ-Al_2_O_3_ at different molar ratios of BDPHP/Ni.

Catalyst	NiMoP (0.0)	NiMoP (0.5)	NiMoP (1.0)
Specific area (m^2^/g)	136	156	164
Pore size (nm)	9.7	9.8	9.9
Total volume (cm^3^/g)	0.33	0.37	0.40

**Table 2:** Percentage of the surface species of freshly sulfided NiMoP/γ-Al_2_O_3_ catalysts.

Catalyst	Synthesis Conditions	Ni 2p Core Level			Mo 3d Core Level	
		NiS	NiMoS	NiOx	MoS_2_(Mo^4+^)	MoOx(Mo^6+^)
NiMoP (0.0)	BDPHP/Ni = 0.0	14	49	37	70	30
NiMoP (0.5)	BDPHP/Ni = 0.5	31	45	24	86	14
NiMoP (1.0)	BDPHP/Ni = 1.0	27	46	27	87	13

**Table 3:** Mo and Ni surface atomic ratios and sulfidation degree of NiMoP/γ-Al_2_O_3_ sulfide catalysts.

Sample	C/Al	Mo/Al	Ni/Al	S/Al	S/(Mo + Ni)	Ni + Mo/Al
NiMoP (0.0)	-	0.07	0.076	0.08	1.07	0.07
NiMoP (0.5)	0.63	0.07	0.020	0.13	1.31	0.09
NiMoP (1.0)	1.00	0.12	0.020	0.15	1.08	0.13

**Table 4:** Conversion and reaction rate constant at steady state (12 h) in the hydrodesulfurization of straight-run gas oil over NiMoP/γ-Al_2_O_3_ sulfide catalysts.

Catalyst	Ratio (BDPHP/Ni)	Conversion (%)	Raman Ratio	k_hds_ (Sw %)^−0.5^ h^−1^
NiMoP (0.0)	0.0	94.1	0.49	6.8
NiMoP (0.5)	0.5	96.7	0.75	9.6
NiMoP (1.0)	1.0	94.5	0.61	7.0

Reaction conditions were T = 340 °C; P = 56 kg/cm^2^; reaction time 12 h; fixed bed reactor.
